# Bilateral Subdiaphragmatic Vagal Nerve Stimulation Using a Novel Waveform Decreases Body Weight, Food Consumption, Adiposity, and Activity in Obesity-Prone Rats

**DOI:** 10.1007/s11695-023-06957-w

**Published:** 2023-12-02

**Authors:** Monique Leinen, Elise F. Grandy, Lourdes M. Ubeira Gebel, Tahimi Machin Santana, Amanda L. Rodriguez, Sundip K. Singh, Michael I. Fernandez, Justin C. Dalugdug, Elaine M. Garcia-Colon, Kamela Lybeshari, Daniel R. Alexander, Maria I. Maura, Maria D. Cabrera Gonzalez, Caroline De Paula Cunha Almeida, Samuel Anyaso-Samuel, Somnath Datta, Matthew A. Schiefer

**Affiliations:** 1 Brain Rehabilitation Research Center, Malcom Randall VA Medical Center, 1601 SW Archer Rd, Gainesville, FL 32608 USA; 2 Department of Biostatistics, University of Florida, 2004 Mowry Rd, 5Th Fl, Gainesville, FL 32603 USA; 3 Department of Biomedical Engineering, University of Florida, 1275 Center Dr, Gainesville, FL 32611 USA

**Keywords:** Vagal nerve stimulation, Obesity, Obesity-prone Sprague Dawley rat

## Abstract

**Introduction:**

Obesity affects millions of Americans. The vagal nerves convey the degree of stomach fullness to the brain via afferent visceral fibers. Studies have found that vagal nerve stimulation (VNS) promotes reduced food intake, causes weight loss, and reduces cravings and appetite.

**Methods:**

Here, we evaluate the efficacy of a novel stimulus waveform applied bilaterally to the subdiaphragmatic vagal nerve stimulation (sVNS) for almost 13 weeks. A stimulating cuff electrode was implanted in obesity-prone Sprague Dawley rats maintained on a high-fat diet. Body weight, food consumption, and daily movement were tracked over time and compared against three control groups: sham rats on a high-fat diet that were implanted with non-operational cuffs, rats on a high-fat diet that were not implanted, and rats on a standard diet that were not implanted.

**Results:**

Results showed that rats on a high-fat diet that received sVNS attained a similar weight to rats on a standard diet due primarily to a reduction in daily caloric intake. Rats on a high-fat diet that received sVNS had significantly less body fat than other high-fat controls. Rats receiving sVNS also began moving a similar amount to rats on the standard diet.

**Conclusion:**

Results from this study suggest that bilateral subdiaphragmatic vagal nerve stimulation can alter the rate of growth of rats maintained on a high-fat diet through a reduction in daily caloric intake, returning their body weight to that which is similar to rats on a standard diet over approximately 13 weeks.

**Graphical Abstract:**

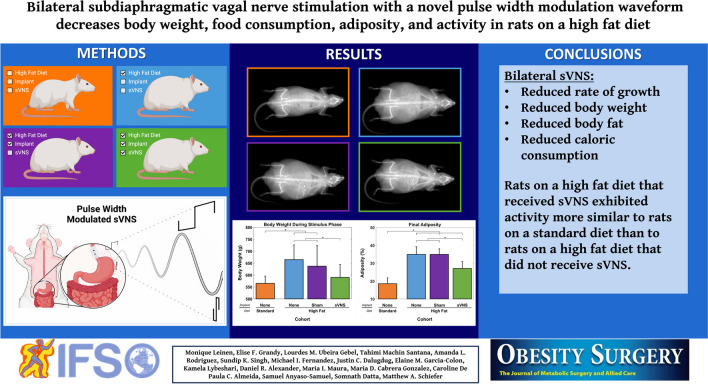

**Supplementary Information:**

The online version contains supplementary material available at 10.1007/s11695-023-06957-w.

## Introduction

In the USA, one in three adults are obese, defined as having a body mass index (BMI) ≥ 30 kg/m^2^ [1]. In a worsening trend, 31 states have obesity rates ≥ 30% and none are below 20% [2]. From 2008 to 2018, the USA saw a year-over-year increase in these high obesity rates of 23%. Obesity is a top priority that, left unaddressed, poses a significant threat to military readiness and national security [3] and a 2017 Pentagon report noted that > 20% of Americans aged 17–24 are ineligible to serve in the US Military due to obesity [4]. Beyond the healthcare crisis of obesity, the economic costs of obesity are also staggering. Obesity-related expenses are estimated to be 20% of annual US healthcare expenditures [5].

While diet and exercise can reduce excess body weight (EBW), 30–60% of individuals relapse [6, 7]. As such, approximately 230,000 Americans undergo bariatric surgery annually [8]. Surgical gastric banding has the lowest mortality rate but highest long-term failure rate [9]. Roux-en-Y gastric bypass (RYGB) and sleeve gastrectomy have lower failure rates but higher mortality rates [9–11]. Intragastric balloons are less invasive but also less effective than RYGB [12]. All of these techniques decrease the stomach volume available for food, which increases stomach wall stretch when food is consumed.

The vagal nerves convey the degree of stomach fullness to the brain via afferent visceral fibers. Most vagal fibers are sensory afferents and nearly all abdominal afferents in the vagus nerves are unmyelinated, including stretch-sensitive mechanoreceptors in the walls of the stomach [13, 14]. As the stomach stretches during food intake, the discharge rate of these mechanoreceptors increases [15–19]. However, studies also indicate that the vagal response to distension is reduced in animals that have been maintained on a high-fat diet [20–22].

The FDA approved vagal nerve stimulation (VNS) to treat intractable epilepsy in 1997 and chronic depression in 2005. There are ongoing investigations into the use of VNS to treat other conditions, including migraine headache, cardiac rhythm, anxiety, Alzheimer’s, fibromyalgia, tinnitus, post-traumatic stress disorder, and inflammation. A reported side effect of VNS has been a reduction in EBW, BMI, and/or adiposity [23, 24]. However, these reports were retrospective in nature and the studies were not designed to assess the effect of VNS on weight loss. Reduction in EBW has motivated several VNS approaches that vary in target location and stimulus parameter selection.

Several studies have found that VNS promotes reduced food intake, causes weight loss, and reduces cravings and appetite [15, 25–33]. VNS has been associated with reduced plasma glucose, cholesterol, visceral fat, and blood pressure [34]. Studies have shown reduced food intake during VNS [32, 35–38]. Recently, an elegant study utilizing optogenetics suggested that activation of axons that innervate stomach and intestinal stretch mechanoreceptors is the primary factor that reduces food consumption [39]. However, it is also worth noting that other studies have found no effect of VNS on body weight, either in animals or in humans [27, 40–45]. For further review of the literature, including the specific location at which VNS was applied, the reader is directed to [35] and [46].

In this study, we investigate the use of a novel VNS waveform on body weight, food consumption, adiposity, and activity. Studies were conducted in obesity-prone Sprague Dawley rats, and we compare outcomes between the treatment group—rats on high-fat chow that received VNS—and three control groups: (1) rats on standard chow that did not receive an implant; (2) rats on high-fat chow that received an implant but no stimulation (sham); and (3) rats on high-fat chow that did not receive an implant. Results showed that rats on a high-fat diet that received subdiaphragmatic vagal nerve stimulation (sVNS) attained a similar weight to rats on a standard diet due primarily to a reduction in daily caloric intake. Rats on a high-fat diet that received sVNS had significantly less body fat than other high-fat controls. Rats receiving sVNS also began moving a similar amount to rats on the standard diet.

This study adds to the scientific body of knowledge in three ways. First, it uses a novel stimulus paradigm in which the pulse width is a function of time. To date, most studies use a static stimulus. That is, each stimulus pulse is identical in amplitude and duration, presented at the same rate. One study that investigated the use of dynamic stimuli found a significant reduction in the consumption of high-fat and high-carbohydrate chow [32]. In this study, we investigate the use of a novel VNS waveform that was adapted from one that was found to produce a sensation of pressure in the phantom fingers of upper extremity amputees [47–49]. It was chosen because the conveyance of information regarding intragastric pressure by vagal axons may be analogous to the conveyance of pressure on fingertips by upper extremity nerves. To our knowledge, no other study has used this type of stimulus for VNS, regardless of target condition. Second, this study is the only VNS study that has analyzed body weight, growth trends, food consumption, adiposity, and daily activity in a single study. Within the field, activity is rarely reported and when it is reported, it is typically anecdotal. In this study, we exhaustively quantified and analyzed nearly 62,500 h of video. Third, most studies only allow 2 weeks for post-surgical healing and the average duration of stimulation is approximately 6 weeks. This study at least doubles both of those durations: post-surgical healing was 4 weeks and the duration of VNS was approximately 13 weeks. Studies often do not last this long because of technical issues. Often, there are failures with externalized electrode lead wires. In our Supplement, we provide details as to how we were able to train our rats to wear vests and use a percutaneous approach.

## Methods

### Rat Husbandry

Charles River Labs initially bred the rats, but they discontinued the animal model during the study. Subsequently, the animal research facility at the LSCVAMC and, later, the MRVAMC and the University of Florida established and maintained a breeding colony. Rats from the breeding colony that were not selected for this study were made available for adoption or to other researchers for their studies.

Rats were maintained on a standard 12:12 light to dark cycle (lights on 6 AM–6 PM). Rats were housed individually in custom large, clear acrylic cages measuring approximately 45 × 45 × 40 cm (width, depth, height). Cages were cleaned and bedding was replaced weekly. At the time of cage cleaning, the rat was weighed. Water and food were provided ad libitum. Rats were assigned to one of 4 groups (see Table 1): a treatment group that received sVNS and was kept on a high-fat diet (Envigo Teklad TD.06414), a control group that received an implant but no sVNS and was kept on a high-fat diet (“sham”), a control group that did not receive an implant and was kept on a high-fat diet, or a control group that did not receive an implant that was kept on a standard diet (Envigo Teklad 2018). Whichever diet the rat was assigned to, the rat started on that diet upon enrollment in the study at 8 weeks of age. Total food consumption was determined by weighing the food introduced to the cage and the food removed from the cage. Any food that was spilled during weighing was not re-introduced and this loss was accounted for during the subsequent food consumption calculation.

### Surgical Implant

Rats underwent surgery around day 110. Three days prior to the surgery, enrofloxacin was added to the drinking water. A day before surgery, meloxicam was administered orally or through subcutaneous injection. The night before surgery, all food was removed from the cage and replaced with a calorie-dense gel to ensure the rat’s stomach was relatively empty during surgery, which facilitated placing the electrode.

On the day of surgery, the rat was anesthetized with 3% isoflurane. Throughout anesthesia, the rat was kept on a warming table (37 °C) to maintain body temperature. Planned incision sites were marked with a surgical marker and areas around the anticipated incision sites were prepared for aseptic surgery.

Throughout anesthesia, heart rate, breathing rate, and blood oxygenation were monitored and recorded at 1 Hz using a MouseOx Plus pulse oximeter (Starr Life Science) via an infrared clip attached to the hind paw. To begin the surgery, an abdominal incision approximately 8 cm in length was made along the linea alba. The abdominal muscle and skin were tented, and the stomach was gently separated from the mesentery. All lobes of the liver anterior to the esophagus were gently raised to reveal the esophagus. A ligation instrument was used to create a tunnel under (posterior to) the esophagus through which the cuff would pass. A custom, adjustable, sterile silicone cuff (Ardiem Medical) was wrapped around the distal esophagus, taking care not to include any paraesophageal fat between the cuff and the esophagus. The cuff contained a pair of bipolar electrodes, had a nominal 4 mm diameter, and was 4 mm long. A small absorbable polydioxanone (PDS II) suture was placed through the silicone walls of the cuff to lock its diameter.

The leads from the cuff were passed through the abdominal muscle via a small lateral incision that was then closed with a PDS II purse string suture. The abdominal muscles were closed with a PDS II simple interrupted suture. Using long curved forceps, a subcutaneous tunnel was created from the abdomen to a midpoint between the abdomen and the planned interscapular exit site. A small incision was made at this midpoint and the leads were routed through the subcutaneous tunnel, leaving a loop in the leads to reduce the risk of tension transmission to the cuff should the leads be pulled. This process was repeated between the midpoint and the exit site, where the lead wires were externalized. All skin incisions were closed with non-absorbable simple interrupted suture using 4–0 Prolene. Additionally, “finger trap” (“Roman sandal”) sutures were used to anchor the percutaneous leads to the skin on the back.

Following surgery, the rats were returned to their cages using softer surgical Alpha-dri bedding (SSP). A heating pad was placed under the cage and maintained at 37 °C to reduce heat loss. Meloxicam continued to be administered for 7 days. Enrofloxacin continued to be administered through drinking water for 10 days. The sutures were removed approximately 11 days after surgery. Stimulation started 4 weeks after surgery.

### sVNS

Bilateral sVNS began approximately 4 weeks after surgery and continued for approximately 90 days. A bipolar, charge-balanced, biphasic, cathodic-first stimulus was applied to the rostral electrodes while the caudal electrodes served as returns. Stimulus was delivered for 12 h per day between the hours of 6:00 PM and 6:00 AM during lights-out. The pulse amplitude was 3 mA and the stimulus frequency was 30 Hz. The pulse width was varied between 10 and 800 μs using a sinusoidal envelope with a frequency of 0.1 Hz (6 cycles per minute). The stimulus was on 30 s, followed by off for 300 s. Stimulus was delivered with the Tucker-Davis Technologies (TDT) IZ2-MH, controlled by the TDT RZ6 using custom-built software in Synapse (TDT) and Matlab (MathWorks). The stimulus was delivered to the rat via a tether from the stimulator to the percutaneous leads. A commutator (slip ring) was positioned between these two points, centered with the cage and positioned approximately 20 cm above the cage to prevent the leads from twisting. Control rats were tethered to mimic the stimulus cable that was connected to rats in the sVNS group.

### Motion Tracking

A closed-circuit camera was mounted above every cage and used to continuously record the activity of the rat using network video recorder (NVR, Lorex). Time-stamped video was recorded in black and white during the day and under infrared illumination (“night vision”) when the lights in the room were off. To reduce total video storage, the frame rate of the video system was reduced to 5 Hz. The Lorex system saved videos in ASF format, which was then converted to MP4 using FFMPEG in PowerShell.

EthoVision (Noldus) was used to quantify the location of the rat in recorded video. Briefly, EthoVision located the rat by distinguishing it from the environment by using a grayscale thresholding algorithm combined with dilations and erosions. EthoVision settings were systematically set to maximize the likelihood that the rat was correctly identified in videos. Because videos were recorded under two lighting conditions, the grayscale range for rat detection was different for daytime videos and nighttime videos. Specifically, the grayscale range for detecting the rat when the lights were on was typically 121–168 (out of 255) while that for when the lights were off was typically 122–153. Videos at transition times between lights on and lights off (6 AM, 6 PM) were excluded. Additionally, a range of acceptable pixel area was set so that neither very small areas nor very large areas could accidentally be misidentified as the rat. The (*x*, *y*) location of the center of the identified rat relative to the lower left corner of the cage (arbitrarily set to (0, 0)) was exported to an Excel file for later analysis. To reduce jitter in the recorded location, lowess smoothing was used.

### DEXA

At the conclusion of the study, the total body adiposity was determined by conducting a dual-energy X-ray absorptiometry (DEXA) InAlyzer scanner (MicroPhotonics). To scan the rat, it was anesthetized using isoflurane, typically at 2.5–3.0%, and positioned prone inside the scanner. A 2-min, live animal scan was collected during which respiration was monitored. Following the DEXA scan, that rat was either humanely euthanized with an overdose of Euthasol (pentobarbital sodium and phenytoin, Virbac) or, if not implanted, made available for adoption.

### Data Analysis

An analysis of variance (ANOVA) was used to compare body weight and daily food/energy consumption across the different populations during the stimulation phase, or the age-matched period for rats that did not receive sVNS. Similarly, an ANOVA was used to compare body weight and daily food/energy consumption across the different populations during the 28-day baseline period prior to surgery, or the age-matched period for rats that did not undergo surgery. Likewise, this approach was used to analyze the adiposity obtained from DEXA scans at the end of the study. For all analyses, a Tukey multiple comparison test was conducted to determine if there was any difference between the groups. A *p*-value < 0.05 suggested a significant difference.

For motion analysis, the day-to-day distance moved was compared between the four groups. Data from daytime videos was analyzed independent of videos from nighttime videos because stimulus was only applied at night. Data from the baseline period was analyzed independent of the videos during the stimulus phase. To analyze the data, we modeled the non-linear relationship between the motion profiles and time using generalized additive models with random effects. For the fixed part of the model, we used thin-plate regression splines to estimate smooth functions of the time trend, while we used random basis splines for the varying smoothed trend for each rat. A generalized likelihood ratio test is employed to test the null hypothesis that the smoothing function for each group is the same. A *p*-value < 0.05 suggested a significant difference in the estimated smooth trends.

## Results

A total of 51 rats were used in the study. Of these, eight were removed from the study: two that became ill during the study and six due to disruptions associated with COVID-19. The remaining 43 rats were divided according to Table 1. At the conclusion of the study, 9 control rats were adopted out as pets. The average age of surgery for rats in the sVNS group was 108 days while the average age of surgery for rats in the sham group was 113 days. There was no significant difference in the age of surgery between these two groups (*p* = 0.6218, two-tailed *t*-test, *t* = 0.5037, df = 15). Data and figures for baseline data are provided in the Supplement.

**Table 1:** Number of rats in each group in the study

Type	Diet	Implant	sVNS	*N*
Control	Standard	No	No	9
Control	High fat	No	No	17
Control	High fat	Yes	No	6
Treatment	High fat	Yes	Yes	11

### Body Weight

**Fig. 1:**
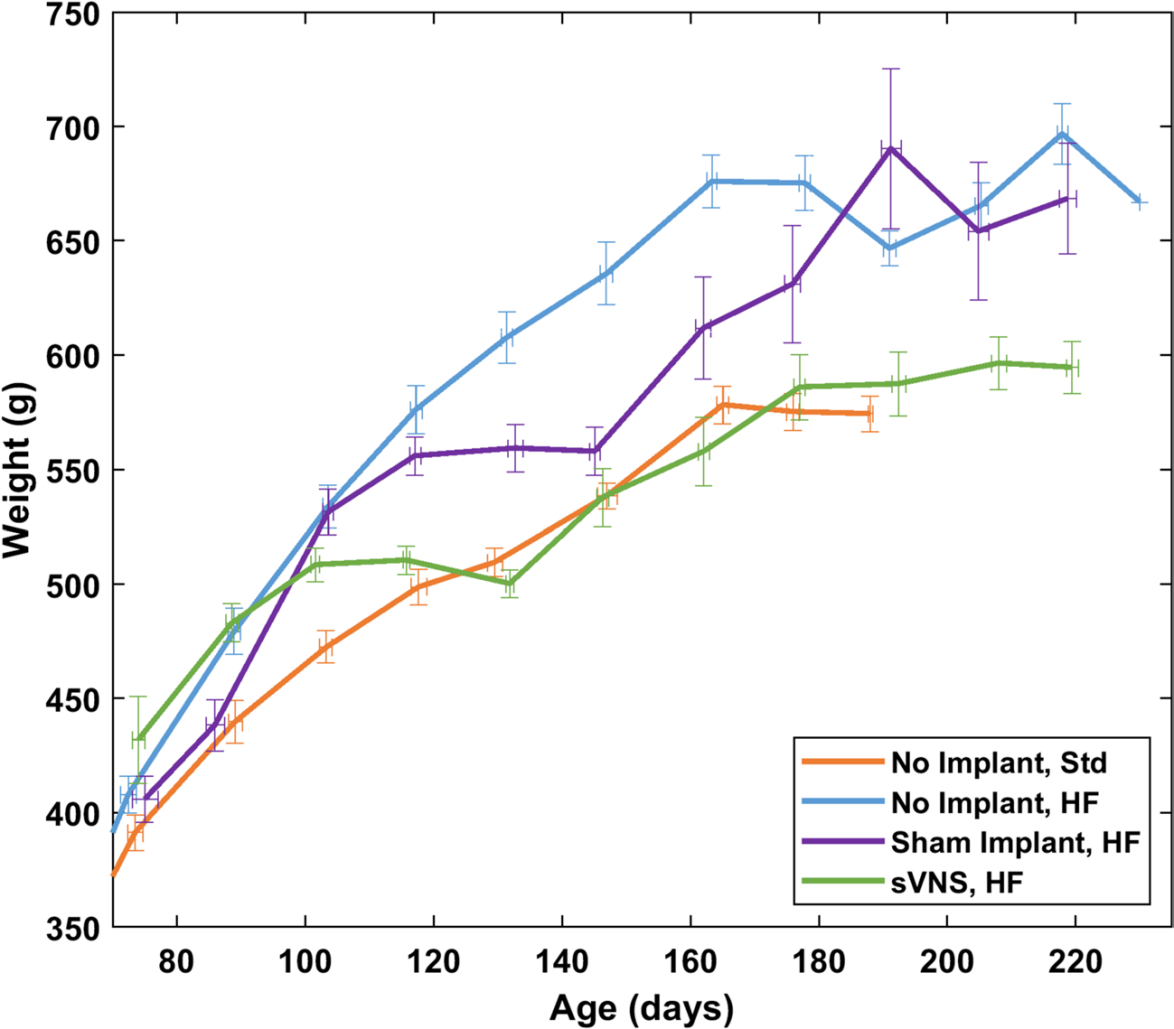
Average growth curves for rats in each of the four groups over time, spanning prior to the baseline period through the stimulation phase. Error bars are standard error of the mean

Body weight for each rat was tracked over time. Data revealed that the growth curve for rats receiving sVNS transitioned from the more rapid rate of growth seen in the two control groups on a high-fat diet to the slower rate of growth seen in the control group on a standard diet (Fig. 1). For the four groups, body weight was similar prior to surgery. During the 28-day baseline period prior to surgery, there was no significant difference in the body weight of rats kept on a high-fat diet. Rats on the high-fat diet had a significantly greater body weight than rats kept on a standard diet. Rats that underwent surgery exhibited a transient plateau or even a decrease in their body weight following surgery that recovered within the 28-day recovery period.

**Fig. 2:**
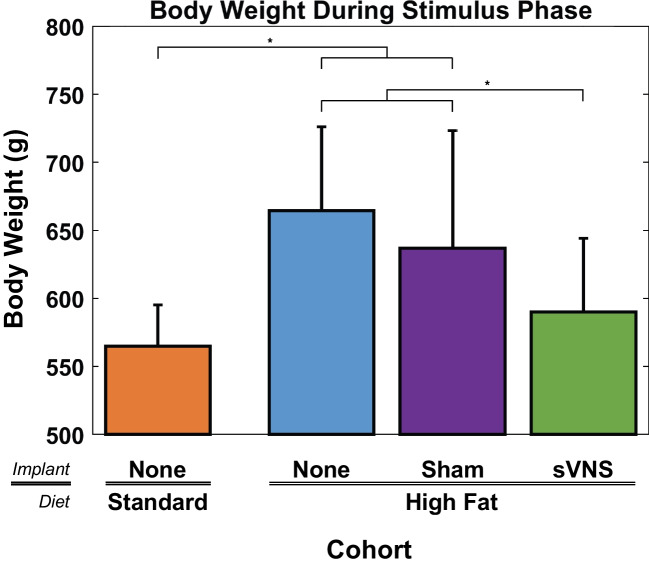
Body weight during the stimulus phase of the four cohorts. Error bars are standard deviation

As time advanced during the stimulation phase, the difference in body weight between the sVNS group and the two control groups on the high-fat diet became more pronounced and significantly different (ANOVA, *p* < 0.001, *F* = 6.91, df_1_ = 3, df_2_ = 283, *R*^2^_adj_ = 87.95%). Rats receiving sVNS ended the stimulus phase weighing 74.4 g (11.2%) and 47.1 g (7.4%) less than rats on a high-fat diet that received no implant and those that received a sham implant, respectively, both of which were significant (Fig. 2). During the stimulation phase, the average body weight for rats receiving sVNS was 25.2 g (4.4%) greater than the control rats on a standard diet, which was not significant.

### Food and Energy Consumption

**Fig. 3:**
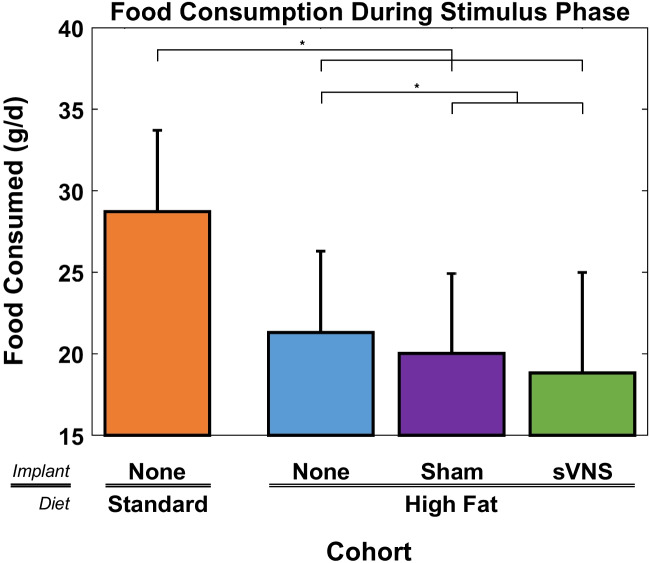
Daily food consumption during the stimulus phase of the four cohorts. Error bars are standard deviation

To determine if reduced weight gain in the sVNS group during the stimulation phase was due, in part, to decreased food/energy intake, the daily food consumption was investigated. Rats on a standard diet consumed a significantly greater amount of food per day than rats on a high-fat diet (Fig. 3) (ANOVA, *p* < 0.001, *F* = 28.97, df_1_ = 3, df_2_ = 1324, *R*^2^_adj_ = 87.55%). On average, rats on a high-fat diet consumed 9.5 g/day (32.2%) less than those on the standard diet. This was also true during the baseline phase (see Supplement). Further, rats receiving sVNS consumed 2.3 g/day (10.9%) less high-fat diet than control rats on the high-fat diet without an implant, which was significant. Rats receiving sVNS consumed 1.2 g/day (6.2%) less than sham rats, although this difference was not significant.

**Fig. 4:**
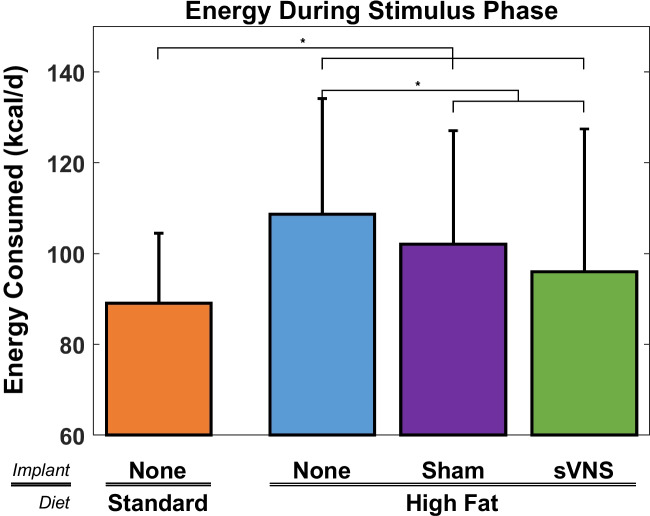
Daily energy consumption during the stimulus phase of the four cohorts. Error bars are standard deviation

While the volume of food consumed by rats on a high-fat diet was significantly lower than that consumed by rats on a standard diet, the same was not true for the amount of energy consumed. Because the high-fat food was calorie-dense, each gram of high-fat diet provided the rat with 65% more calories than each gram of standard diet chow. As such, rats on a standard diet consumed significantly less calories per day than rats on high-fat diets (Fig. 4) (ANOVA, < 0.001, *F* = 32.56, df_1_ = 3, df_2_ = 1324, *R*^2^_adj_ = 84.65%). Rats receiving sVNS consumed 4.4 kcal/day (4.8%) more than rats on the standard diet and 11.7 kcal/day (10.9%) less than control rats on a high-fat diet that were not implanted, both of which were significant.

### Adiposity and Lean Tissue

**Fig. 5:**
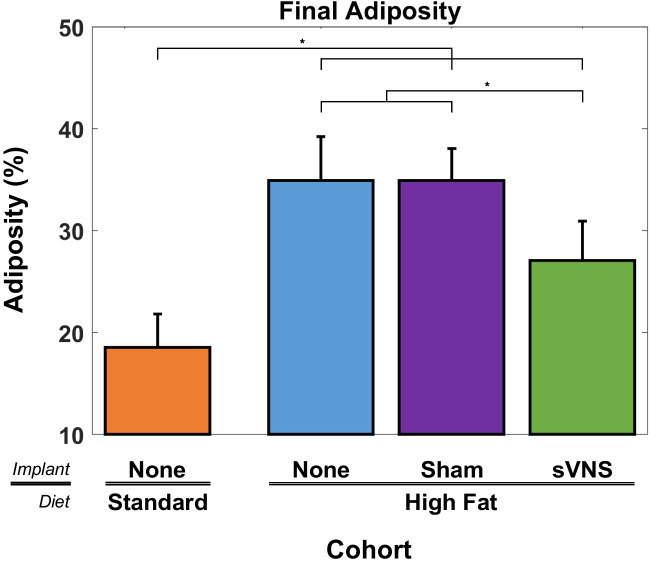
Adiposity (percent body fat) as determined by DEXA scans at the end of the study for each cohort. Error bars are standard deviation

**Fig. 6:**
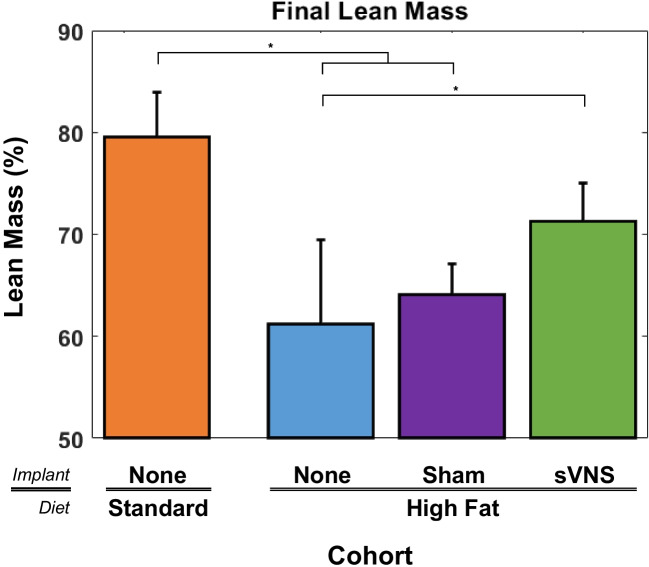
Lean Tissue as determined by DEXA scans at the end of the study for each cohort. Error bars are standard deviation

At the conclusion of the study, each rat was scanned with a DEXA scanner to quantify whole-body adiposity and lean tissue. The adiposity of control rats on a standard diet was significantly less than that of all cohorts on the high-fat diet (Fig. 5) (ANOVA, *p* < 0.001, *F* = 21.53, df_1_ = 3, df_2_ = 30, *R*^2^_adj_ = 67.24%). However, rats receiving sVNS exhibited a 22.6% reduction in adiposity, dropping from 34.9 to 27.0% compared to control rats on a high-fat diet regardless of the presence of an implant, which was significant. Likewise, lean body mass was significantly different between groups (Fig. 6) (ANOVA, *p* < 0.001, *F* = 13.70, df_1_ = 3, df_2_ = 30, *R*^2^_adj_ = 55.95%). While rats on a standard diet had the highest percentage of lean tissue, it was not significantly different from rats that received sVNS and both groups had significantly more lean tissue than control rats on the high-fat diet.

### Motion Tracking

**Fig. 7:**
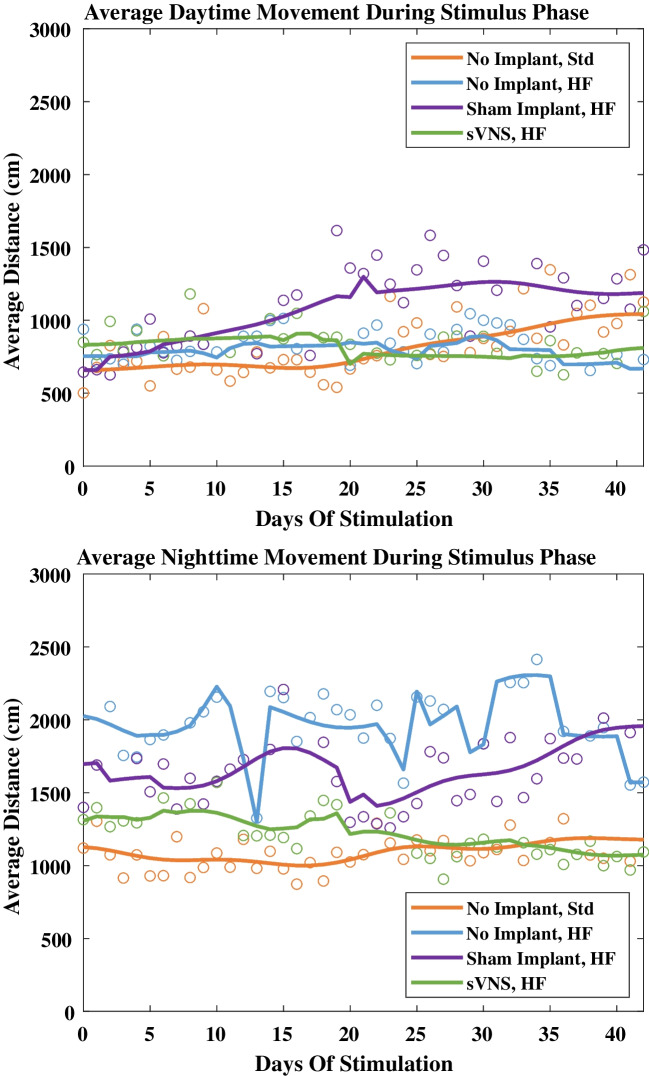
Average distance moved during each 30-min video during the day (top plot) or night (bottom plot) during the stimulus phase. Data points are averaged across all rats. Trend lines are the average of the predicted mean distance moved by the rats in each group

Approximately 62,500 h of video (nearly 125,000 video files) was analyzed using EthoVision to quantify the (*x*, *y*) location of the rat within its cage. The day-to-day trends in the average distance moved were then analyzed to determine if different cohorts appeared to behave differently, as defined by the smoothing function fit to their motion data. During the stimulus phase and while lights were on, the sham cohort behaved differently than the control cohort on a standard diet, which itself did not behave differently than the other two cohorts (generalized likelihood ratio test, *p* < 0.05, *R*^2^_adj_ = 60.7%). Video analysis revealed that the sham cohort tended to move a greater distance during the day throughout the latter half of the stimulus phase (Fig. 7, top). However, when the lights were off, none of the rats on high-fat diets behaved like those on the standard diet (generalized likelihood ratio test, *p* < 0.05, *R*^2^_adj_ = 87.1%).

## Discussion

As food transits through the digestive system, stomach and intestinal wall stretch activates intraganglionic laminar ending (IGLE) mechanoreceptors, which contributes to a sensation of satiety and a reduction in food consumption [50]. This has been demonstrated through experiments using gavage [51], ligation [19], and optogenetic activation [39] as well as several studies using electrical nerve stimulation. However, studies suggest that animals exposed to a high-fat diet have less vagal tone during stomach distension, which may promote overeating [15, 18–21, 52, 53]. Thus, the decision to use vagal nerve stimulation to increase vagal tone and convey a sense of fullness has been studied by several groups. Looking across the results from this study, the data shows that the sVNS group trended toward lower body weight and adiposity due to lower caloric intake but not likely due to an increase in activity that would result in increased energy expenditure nor due to a cessation of activity that would suggest discomfort.

During the baseline phase, all rats on a high-fat diet weighed significantly more than those on the standard diet. However, rats receiving sVNS attained a body weight that was greater than but not significantly different from rats kept on a standard diet. By the end of the study, rats receiving sVNS weighed approximately 595 g, compared to shams that weighed approximately 670 g. While the adiposity of rats on the high-fat diet was significantly greater than those on the standard diet, it is worth noting that the adiposity of rats receiving sVNS was significantly less than all other rats on the high-fat diet and the lean body mass was not significantly different between the group receiving sVNS and the control group on the standard diet. The reduction of adiposity found in this study was similar to the 20% reduction in visceral fat demonstrated in obese Wistar rats after 12 weeks of cervical VNS [34]. Results further suggest that the activity of rats receiving sVNS becomes more similar to rats on a standard diet than sham or non-implanted rats on a high-fat diet.

In terms of rate of weight gain between the start and end of the study, the sVNS group exhibited a 38% increase in body weight, whereas rats on the standard diet exhibited a 47% increase in body weight and rats with and without an implant on a high-fat diet exhibited a 65% and 64% increase in body weight, respectively. Therefore, the sVNS group gained weight at a lower rate than any of the other groups. This is similar to the approximately 41% and 59% weight gains observed in pigs that did and did not receive bilateral sVNS for 12 weeks using a similar stimulus paradigm, respectively [54]. When comparing the sVNS group to either of the control groups on the high-fat diet, the sVNS group exhibited a 42% decrease in weight gain, which is similar to the 48% reported during bilateral thoracic VNS in pigs [55]. While surgery and/or the implant in the sham population did reduce body weight compared to the control cohort on the high-fat diet that did not receive an implant, the reduction in body weight was transient and the two populations ended the study with similar weights and weight gains.

All rats on the high-fat diet consumed significantly less food than those on the standard diet. Although the weight of food consumed by the high-fat cohorts was less than that consumed by the cohort on a standard diet, due to the caloric density of the food, the rats on the high-fat diet consumed significantly more calories per day than those on the standard diet. This was true both during baseline and stimulus phases. Results also reveal that rats with an implant consumed significantly less food and, hence, significantly fewer calories than the cohort on a high-fat diet that did not receive an implant. Because there was a reduced caloric consumption by the sham group, we can reasonably assume that some of the reduced caloric consumption by the sVNS group was due to surgery or the implant and not due to sVNS. However, the sVNS group exhibited a further reduction in caloric intake, indicating that sVNS was effective at curtailing food consumption.

To our knowledge, this is the first study to exhaustively quantify movement and attempt to determine if there was a statistical difference in day-to-day patterns of movement between the different cohorts. Results confirm that rats receiving sVNS do not appear to behave abnormally and that the reduction in food consumption observed during sVNS is unlikely linked to lethargy or sequestration. Given the complexity of the statistical analyses, only the control group on a standard diet was compared to the other three groups. Pairwise comparisons were not conducted because corrections for multiple comparisons and the interpretation of the results were exceptionally esoteric. The results suggested that the sham group behaved differently from the control group on a standard diet during the day. Specifically, the sham group tended to move more during the day as the study progressed. It remains uncertain if this difference was meaningful, but, in general, all four cohorts moved similar amounts and exhibited similar behavior during the day. The same was not true at night.

During the night, all rats tended to be more active, though this was most pronounced in rats on the high-fat diet, resulting in a clearer difference in the different cohorts’ patterns of behavior. There were some interesting observations that came out of the data. Non-implanted rats on the high-fat diet tended to move more than any other cohort. We do not know why this occurred. It may be that this cohort was driven to forage for more food, but we did not distinguish between foraging behavior and other types of motion during the video analyses. Near the beginning of the stimulus phase, rats on the high-fat diet that were implanted moved similar amounts, but over time, these cohorts diverged and sham rats slowly increased their distance moved while sVNS rats decreased their distance moved. By the end of the study, non-implanted rats on the high-fat diet and those in the sham group were travelling similar distances. Likewise, rats on the standard diet and rats receiving sVNS were travelling similar distances, which were less than the distances traveled by the other two cohorts.

While the use of VNS to treat obesity is not new, the waveform used in this study is novel within the field of VNS. The decision to use this waveform stemmed from results in a prior study [49]. In that study, which focused on the tactile system, altering the pulse width on a pulse-by-pulse basis using a sinusoidal shaping envelope at a naturally occurring frequency produced a sensation of pressure in the phantom hand of upper extremity amputees. In [49], a frequency of 1 Hz was chosen, aligning with an average human heart rate. In this study, 0.1 Hz was chosen to align with the typical rate of antral peristalsis in rats. Because the pulse width varied between 10 and 800 μs and given the higher threshold associated with unmyelinated fibers that comprise the majority of axons in the subdiaphragmatic vagus nerves [46], the portions of the stimulus at lower pulse widths were below activation threshold while those at the larger pulse widths were above activation threshold. Computer simulations suggest that the nerves were “paced” at 0.1 Hz but with a dynamically activated (i.e., not static) population of axons on a pulse-by-pulse basis [56]. While the specific dynamic stimulus waveform used in this study is novel, another study has used dynamic waveforms. Using 30 Hz, 30-s-on, 5-min-off bilateral sVNS in pigs, Malbert et al. demonstrated that pulsons decreased daily energy consumption, particularly of high-fat diet, whereas both pulsons and more traditional sVNS reduced consumption of a high-glucose diet [32]. In a recent study, Debelle et al. demonstrated that using a dynamic and adaptive stimulation reduced food intake and hence caloric intake, ultimately reducing body weight in dogs [38].

One of the challenges within the field is that there are several VNS studies that have reported a range of results on body weight, weight gain, food consumption, adiposity, and various metabolic metrics, but these studies have used a wide range of stimulus parameters (current-controlled, voltage-controlled, varying duty cycles, varying pulse widths), target stimulus locations (cervical, thoracic, subdiaphragmatic, unilateral or bilateral), duration of stimulation (ranging from a few days to nearly a year), animal models (rats, rabbits, dogs, pigs), and animal ages. There is little consistency between these studies, making it very difficult to compare results between studies. Our study used Sprague Dawley rats that underwent surgery around day 110 and started receiving bilateral sVNS around day 140 for 90 days until 230 days of age. The sVNS was on for 30 s, off for 300 s, but only applied at nighttime. Stimulus was 3 mA at 30 Hz with a time-varying pulse width controlled by a sinusoid that had a frequency of 0.1 Hz. Other studies have used bilateral sVNS in rats [37, 57, 58], but Johannessen et al. is the most comparable. Johannessen et al. applied sVNS at 30 Hz, on for 30 s and off for 300 s. Whereas our study used a 3-mA stimulus with a variable pulse width ranging from 10 to 800 μs, Johannessen et al. used a pulse amplitude that gradually increased from 0.5 to 2.0 mA with a fixed 500-μs pulse width. Another difference was that Johannessen et al. provided up to 56 days of continuous stimulation whereas this study provided 90 days of stimulation that was only on during the night. Johannessen et al. reported that rats receiving sVNS weighed 10% less than shams at the end of the study. By the end of our study, rats receiving sVNS weighed 11% less than the sham group, putting our results in line with those presented with Johannessen et al. Our results were also similar to the 10% difference in body weights observed in pigs receiving bilateral thoracic VNS using stimulation parameters that were nearly identical to Johannessen’s but over a 98-day period [35]. Other studies using similar stimulus paradigms in different animal models, in different target locations, and/or for different durations of time have reported similar amounts of weight loss between their VNS groups and the sham groups [32, 35, 54, 55]. What begins to emerge is the possibility that this stimulus paradigm, while effective at reducing weight, is limited. Given the range of variation in the studies, comprehensive research that maps stimulus input to behavioral output is needed.

Another observation from this study is that the duration of post-surgical recovery provided by many studies may be too short. We found that weight gain in implanted animals did not return until approximately 4 weeks after surgery. This longer recovery time is likely influenced by animal model, age, target location, surgeon, surgical technique, and surgical implant. Studies that initiate VNS before full recovery may be confounded by the effects of surgery if they are not carried out long enough. Therefore, it is important to consider recovery time in the study design.

Finally, it is worth noting that rats in the control group on the standard diet do not appear to have been aged as long as rats in the other three groups (Fig. 1). In fact, 4 rats in the control group were aged significantly longer to 320 days. However, body weights collected between 190 and 270 days of age were lost due to a corrupted file. The 5 remaining rats in this control group that came later in the study were not aged beyond 190 days because there was not sufficient time to age rats to 270 days (when the corrupted data file was fixed) due to COVID disruptions that required adjusting the study timeline.

This study has limitations, the most significant of which was that stimulation of the vagus nerves was not verified by secondary means and, as such, we cannot say with certainty that the sVNS altered nerve activity. Although lead impedance was monitored daily, this only confirmed the health of the implant. Future studies should monitor the animal using secondary measures to confirm that the electrical stimulation activated the vagus nerve. Further, while the outcomes were significant and strongly suggest that electrical stimulation activated the nerves, it is unclear if the population of activated axons was exclusively afferent, exclusively efferent, or a combination of both, the latter of which is most likely. As such, techniques to monitor parasympathetic tone, such as heart rate variability and gastrointestinal transit time, should be considered. Alternatively, periodic monitoring with fMRI or PET could confirm if sVNS affected activity in the brain, particularly food-based and satiety centers such as the ventromedial hypothalamus, dorsal medial nucleus of the hypothalamus, lateral hypothalamus, and ventral tegmental area.

Another limitation of this study comes from using a rodent model. The use of rodents in preclinical studies, including obesity studies, is well-established. However, there are inherent limitations to using animal models. The body weight of Sprague Dawley rats tends to plateau around 18–24 months [59]. Waiting this long before implanting and delivering sVNS carries significant housing expenses, increases the risk of surgical mortality, and provides little time to conduct a longitudinal experiment before the obese rat would likely succumb to old age and comorbidities. As such, studies using younger rats that are still growing are limited to analyzing outcomes in terms of a reduction in weight gain rather than weight loss. This makes it difficult to compare outcomes between preclinical and clinical studies.

Another limitation associated with the use of rodents comes in the form of husbandry. Because there were percutaneous leads from the implant, rats had to be housed individually to protect the leads from damage that would occur during rough play or grooming. Individual housing also allowed practical quantification of daily food consumption and motion tracking. However, rats are social animals and isolation is known to change their behavior. As such, all rats in this study could have been affected by an unaccounted variable: isolation. To try to minimize the effects of isolation, we used large cages that allowed plenty of space to play. The cages were clear acrylic, allowing rats to see each other. Cages were open on the top, allowing rats to smell their neighbors. We also dedicated time each week to handling, petting, and interacting with each rat to provide additional enrichment.

A further limitation is associated with the specific strain of rat used in this study. As noted, these were obesity-prone Sprague Dawley rat from Charles River Labs for consistency with prior studies. Unfortunately, Charles River Labs discontinued this animal model during the study. As a result, we established breeding colonies. However, because there were a limited number of rats to start the colony, the genetic diversity within the colonies diminished over time. It is uncertain if there was any effect on outcomes, but effects should have been minimized by randomizing the cohort to which a rat was assigned.

If the ultimate goal is to translate sVNS to the clinic to treat obesity, then the technology must compete with well-established bariatric surgical treatments as well as the newer semaglutide GLP-1 and tirzepatide GLP-1/GIP agonists. Currently, the weight loss associated with these other methods exceeds weight loss with sVNS, although, as noted above, rodents were still in the growth phase during this study so the comparisons are difficult. This highlights a final limitation of this and other preclinical studies: the sVNS parameters have not been optimized. In general, optimization is a significant challenge because there are three independent stimulus parameters (pulse width, pulse amplitude/voltage, stimulus frequency), all of which can be a function of time, which itself can be applied on a pulse-by-pulse basis (short time scale) as well as used to define an on–off duty cycle (long time scale). There are myriad combinations of stimulus parameters within this multidimensional space. As such, it is likely that preclinical studies will be limited in their success and they may never achieve the weight loss seen with bariatric surgery or drugs. Indeed, it may only be possible for sVNS to achieve better outcomes when descriptive verbal feedback is available to guide stimulus parameter decisions.

The ultimate goal is to successfully translate optimized sVNS to the clinic. There are several future steps that must occur. Secondary verification is essential for translation to clinical trials. Indeed, our current studies include longitudinal fMRI and connectomics to both confirm that sVNS is activating axons and to better understand the effect of varied stimulus waveforms and chronic diet on activated pathways. We are investigating methods to optimize the stimulus parameters with the goal of establishing a standardized protocol that will allow us to set the best stimulus parameters on an individualized basis. Ideally, future studies will allow the animals to be housed together and interact socially. To this end and once there is a smaller range of near-optimal stimulus parameters, the implants should be fully internalized. This includes the cuff and the stimulator. Development of small, hermetically sealed, multichannel implantable pulse generators that support wireless programming, have either an extended battery life or support wireless recharging, and are affordable should be actively pursued. Because rats consume food periodically throughout the day, such a system does not have to be closed-loop. However, when such a system is translated to humans that eat less frequently, the implantable system should be closed-loop and triggered by stomach stretch or food consumption rather than open-loop and required to operate continuously. Finally, finding the best stimulus parameter combinations may not be possible until verbal descriptions can be used to guide parameter choices. To this end, short-term implants that piggyback off of scheduled bariatric surgeries may provide the best pathway to optimization.

## Conclusions

Results from this study suggest that bilateral subdiaphragmatic vagal nerve stimulation can alter the rate of growth of rats maintained on a high-fat diet through a reduction in daily caloric intake, returning their body weight to that which is similar to rats on a standard diet over approximately 13 weeks.

### Supplementary Information

Below is the link to the electronic supplementary material.

Supplementary file1 (DOCX 267 KB)

## Data Availability

The datasets generated and analyzed during the current study will be made available in the Harvard Dataverse repository upon acceptance of the manuscript. The reader may also contact the corresponding author directly by email.

## References

[1] Ogden CL, Carroll MD, Kit BK (2012). Prevalence of obesity in the United States, 2009–2010. NCHS Data Brief.

[2] Adult obesity prevalence maps, Centers for Disease Control and Prevention. 2023; https://www.cdc.gov/obesity/data/prevalence-maps.html.

[3] Cheney SA, Xenakis SN. Obesity's increasing threat to military readiness: the challenge to U. S. national security. American Security Project. JSTOR. 2022; http://www.jstor.org/stable/resrep46869.

[4] Spoehr T, Handy B (2018). The looming national security crisis: young Americans unable to serve in the military. Backgrounder.

[5] Cawley J, Meyerhoefer C (2012). The medical care costs of obesity: an instrumental variables approach. J Health Econ.

[6] Inelmen EM (2005). Predictors of drop-out in overweight and obese outpatients. Int J Obes.

[7] Douketis JD, Macie C, Thabane L (2005). Systematic review of long-term weight loss studies in obese adults : clinical significance and applicability to clinical practice. Int J Obes.

[8] Estimate of bariatric surgery numbers, 2011-2021, American Society for Metabolic and Bariatric Surgery. 2022; https://asmbs.org/resources/estimate-of-bariatric-surgery-numbers.

[9] Madura JA, Dibaise JK. Quick fix or long-term cure? Pros and cons of bariatric surgery. F1000 Med Rep. 2012;4:19. 10.3410/M4-19 PMC3470459 23091563

[10] Gill RS, Birch DW, Shi X (2010). Sleeve gastrectomy and type 2 diabetes mellitus: a systematic review. Surg Obes Relat Dis.

[11] BruschiKelles SM, Diniz MFHS, Machado CJ (2014). Mortality rate after open Roux-in-Y gastric bypass: a 10-year follow-up. Brazilian J Med Biol Res.

[12] Tate CM, Geliebter A (2017). Intragastric balloon treatment for obesity: review of recent studies. Adv Ther.

[13] Falempin M, Mei N, Rousseau JP (1978). Vagal mechanoreceptors of the inferior thoracic oesophagus, the lower oesophageal sphincter and the stomach in the sheep. Eur J Physiol.

[14] Prechtl JC, Powley TL (1990). The fiber composition of the abdominal vagus of the rat. Anat Embryol (Berl).

[15] Peles S (2003). Enhancement of antral contractions and vagal afferent signaling with synchronized electrical stimulation. Am J Physiol Gastrointest Liver Physiol.

[16] Schloithe AC, Woods CM, Davison JS (2006). Pancreatobiliary afferent recordings in the anaesthetised Australian possum. Auton Neurosci Basic Clin.

[17] Kentish SJ, Page AJ (2015). The role of gastrointestinal vagal afferent fibres in obesity. J Physiol.

[18] Ozaki N, Sengupta JN, Gebhart GF (1999). Mechanosensitive properties of gastric vagal afferent fibers in the rat. J Neurophysiol.

[19] Miranda A (2009). Altered mechanosensitive properties of vagal afferent fibers innervating the stomach following gastric surgery in rats. Neuroscience.

[20] Kentish S (2012). Diet-induced adaptation of vagal afferent function. J Physiol.

[21] Kentish SJ (2013). Gastric vagal afferent modulation by leptin is influenced by food intake status. J Physiol.

[22] Kentish SJ, Ratcliff K, Li H (2015). High fat diet induced changes in gastric vagal afferent response to adiponectin. Physiol Behav.

[23] Burneo JG, Faught E, Knowlton R (2002). Weight loss associated with vagus nerve stimulation. Neurology.

[24] Pardo J (2007). Weight loss during chronic, cervical vagus nerve stimulation in depressed patients with obesity: an observation. Int J Obes.

[25] Despa F (2009). Electromuscular incapacitation results from stimulation of spinal reflexes. Bioelectromagnetics.

[26] Ziomber A (2009). Magnetically induced vagus nerve stimulation and feeding behaviour in rats. J Physiol Pharmacol.

[27] Gil K, Bugajski A, Kurnik M (2009). Physiological and morphological effects of long-term vagal stimulation in diet induced obesity in rats. J Physiol Pharmacol.

[28] Wu X, McLaughlin L, Polk JP (2009). A pilot study to evaluate the effect of splanchnic nerve stimulation on body composition and food intake in rats. Obes Surg.

[29] Banni S (2012). Vagus nerve stimulation reduces body weight and fat mass in rats. PLoS One.

[30] Osharina V, Bagaev V, Wallois F (2006). Autonomic response and Fos expression in the NTS following intermittent vagal stimulation: importance of pulse frequency. Auton Neurosci.

[31] Sobocki J, Fourtanier G, Estany J (2006). Does vagal nerve stimulation affect body composition and metabolism? Experimental study of a new potential technique in bariatric surgery. Surgery.

[32] Malbert CH, Bobillier E, Picq C (2017). Effects of chronic abdominal vagal stimulation of small-diameter neurons on brain metabolism and food intake. Brain Stimul.

[33] Malbert CH (2021). Vagally mediated gut-brain relationships in appetite control-insights from porcine studies. Nutrients.

[34] Samniang B (2016). Vagus nerve stimulation improves cardiac function by preventing mitochondrial dysfunction in obese-insulin resistant rats. Sci Rep.

[35] Val-Laillet D, Biraben A, Randuineau G (2010). Chronic vagus nerve stimulation decreased weight gain, food consumption and sweet craving in adult obese minipigs. Appetite.

[36] Roslin M, Kurian M (2001). The use of electrical stimulation of the vagus nerve to treat morbid obesity. Epilepsy Behav.

[37] Laskiewicz J, Krolczyk G, Zurowski D (2003). Effects of vagal neuromodulation and vagotomy on control of food intake and body weight in rats. J Physiol Pharmacol.

[38] Debelle A (2022). Impact of adaptive gastric electrical stimulation on weight, food intake, and food intake rate in dogs. Artif Organs.

[39] Bai L (2019). Genetic identification of vagal sensory neurons that control feeding. Cell.

[40] Shikora SA (2009). Implantable gastric stimulation for the treatment of clinically severe obesity: results of the SHAPE trial. Surg Obes Relat Dis.

[41] Rush AJ (2005). Effects of 12 months of vagus nerve stimulation in treatment-resistant depression: a naturalistic study. Biol Psychiatry.

[42] Koren MS, Holmes MD (2006). Vagus nerve stimulation does not lead to significant changes in body weight in patients with epilepsy. Epilepsy Behav.

[43] Abubakr A, Wambacq I (2008). Long-term outcome of vagus nerve stimulation therapy in patients with refractory epilepsy. J Clin Neurosci.

[44] Kansagra S, Ataya N, Lewis D (2010). The effect of vagus nerve stimulation therapy on body mass index in children. Epilepsy Behav.

[45] Díaz-Güemes I, Sánchez FM, Luis L (2007). Continuous vagus nerve stimulation effects on the gut-brain axis in swine. Neuromodulation.

[46] Pelot NA, Grill WM. Effects of vagal neuromodulation on feeding behavior. Brain Res. 2018;1693, Part B:180–187. 10.1016/j.brainres.2018.02.003 PMC6003853 29425906

[47] Tan DW, Schiefer MA, Keith MW (2015). Stability and selectivity of a chronic, multi-contact cuff electrode for sensory stimulation in human amputees. J Neural Eng.

[48] Schiefer MA, Graczyk EL, Sidik SM (2018). Artificial tactile and proprioceptive feedback improves performance and confidence on object identification tasks. PLoS ONE.

[49] Tan D, Schiefer MA, Keith MW (2014). A neural interface provides long-term stable natural touch perception. Sci Transl Med.

[50] Wang YB, de Lartigue G, Page AJ. Dissecting the role of subtypes of gastrointestinal vagal afferents. Front Physiol. 2020;11:643. 10.3389/fphys.2020.00643 PMC7300233 32595525

[51] Powley TL, Phillips RJ (2004). Gastric satiation is volumetric, intestinal satiation is nutritive. Physiol Behav.

[52] Kentish SJ (2015). TRPV1 channels and gastric vagal afferent signalling in lean and high fat diet induced obese mice. PLoS ONE.

[53] Loper H (2021). Both high fat and high carbohydrate diets impair vagus nerve signaling of satiety. Sci Rep.

[54] Malbert C-H, Picq C, Divoux JL (2017). Obesity-associated alterations in glucose metabolism are reversed by chronic bilateral stimulation of the abdominal vagus nerve. Diabetes.

[55] Biraben A, Guerin S, Bobillier É (2008). Activation centrale a la suite d’une stimulation vagale chronique chez le porc: apports de l’imagerie fonctionnelle. Bull l’Académie Vétérinaire Fr.

[56] Leinen M, et al. Bilateral subdiaphragmatic vagal nerve stimulation (sVNS) using intermittent pulse width modulation reduces weight gain in obesity-prone Sprague Dawley rats exposed to a high fat diet. In: Proceedings from the Society for Neuroscience Annual Conference 2022; San Diego; https://www.abstractsonline.com/pp8/#!/10619/presentation/86048.

[57] Krolczyk G, Laskiewicz J, Sobocki J (2005). The effects of baclofen and the feeding behavior and body weight of vagally stimulated rats. J Physiol Pharmacol.

[58] Johannessen H (2017). Vagal blocking for obesity control: a possible mechanism-of-action. Obes Surg.

[59] Altun M, Bergman E, Edström E (2007). Behavioral impairments of the aging rat. Physiol Behav.

