# Supplementary material for: Bilateral Subdiaphragmatic Vagal Nerve Stimulation Using a Novel Waveform Decreases Body Weight, Food Consumption, Adiposity, and Activity in Obesity-Prone Rats
Source: Obes Surg. 2023 Dec 2;34(1):1–14. doi: 10.1007/s11695-023-06957-w (PMC10781827; doi:10.1007/s11695-023-06957-w)
Supplement: Supplementary file 1 — Supplementary file1 (DOCX 267 KB) [file 11695_2023_6957_MOESM1_ESM.docx]

*Baseline Data*

Below are plots of the body weight, food consumption, energy consumption, and movement during the 28-day baseline period prior to surgery.


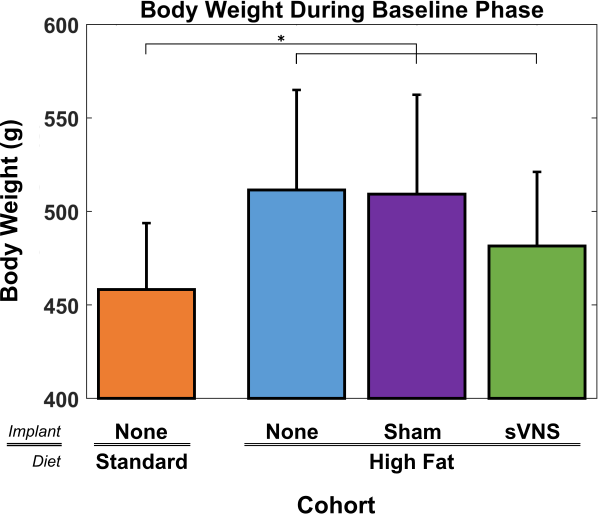


**Figure S1:** There was no significant difference in body weight during the baseline phase between the three cohorts of rats on a high fat diet and all three cohorts had a significantly greater body weight than the cohort on the standard diet (ANOVA, p<0.001, F=32.56, df_1_=3, df_2_=147, R^2^_adj_=72.16%). On average, rats on the standard diet weighed 458±36 g whereas rats on the high fat diet weighed 498±50 g.


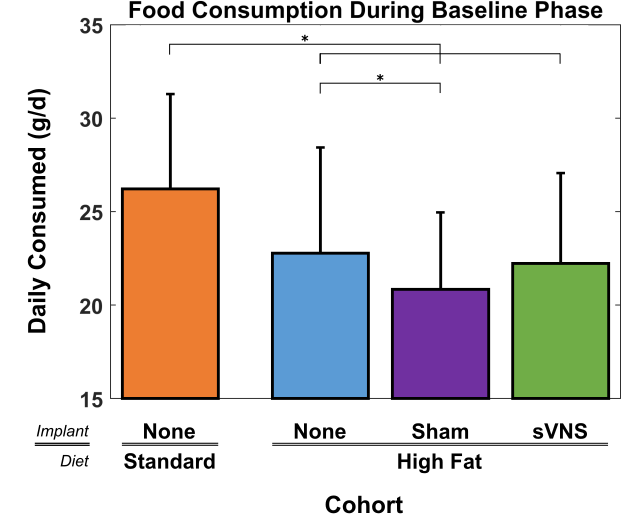


**Figure S2:** Rats on a standard diet consumed significantly more food than rats on a high fat diet during the baseline phase (ANOVA, p<0.001, F=32.56, df_1_=3, df_2_=488, R^2^_adj_=84.70%). On average, rats on the standard diet consumed 26±5 g/d whereas rats on the high fat diet consumed 22±5 g/day.


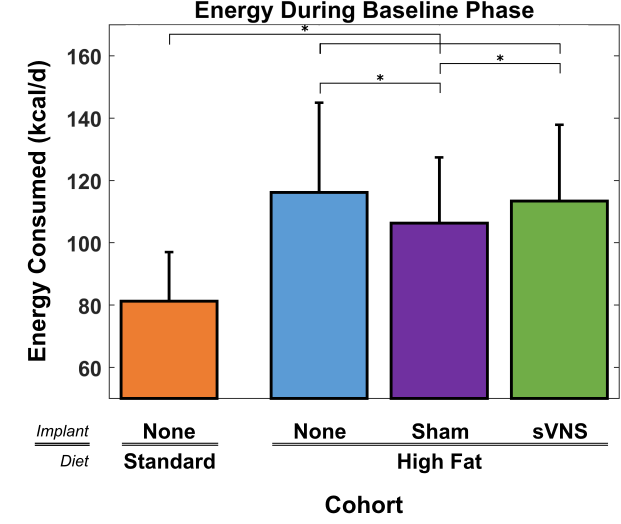


**Figure S3:** Rats on a standard diet consumed significantly fewer calories than rats on a high fat diet during the baseline phase (ANOVA, p<0.001, F=32.56, df_1_=3, df_2_=488, R^2^_adj_=87.00%). On average, rats on the standard diet consumed 81±16 g/d whereas rats on the high fat diet consumed 115±27 g/day.


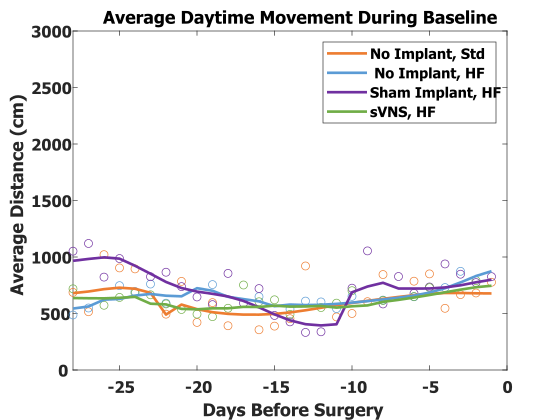

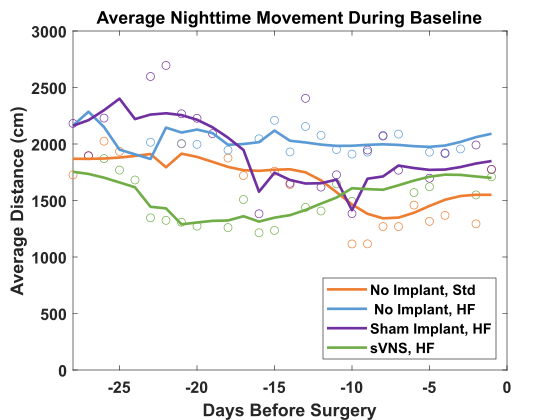


**Figure S4:** Average distance moved during each 30-minute video during the day (top plot) or night (bottom plot) during the baseline phase. Data points are averaged across all rats. Trend lines are the average of the predicted mean distance moved by the rats in each group. During the night, rats in all groups moved more than during the day. As with data from the stimulation phase, rats on the standard diet were found to move differently than rats on a high fat diet (generalized likelihood ratio test, p<0.05, R^2^_adj_=60.0% for daytime data, R^2^_adj_=89.7% for nighttime data). There were no clear patterns of divergence or convergence in behavior during the baseline period.

## *Acclimating Rats to Harnesses*

Prior to vest and tether training, rats were allowed to acclimate to their cages for at least two days. In the next three days, researchers interacted with each rat individually by placing them in a large box containing a blanket and spandex vest (Braintree Scientific, Inc.). Researchers would guide the rats to sniff the vest and become comfortable being around it; positive reinforcement treats (Bio-Serv rainbow foraging bits) were given to encourage this behavior. During this time, the rat was getting used to being handled by the researcher. Once the rat was acclimated to the vest, the researchers placed the vest on the rat either by letting it step through the armhole or by having one person hold the rat while wrapped in the blanket and another person place the vest on the rat. On the first day, the vest is placed on the rat for approximately 5 minutes. If the rat takes the vest off before this time, the vest will not be placed back on. The time the rat spent in the vest increased each day gradually for the next week, beginning at 5-10 minutes, increasing until the vest was worn throughout the workday (approximately 8 hours) and then overnight (about 15 hours). Every time rats took the vest off, if there weren’t any signs of distress, the vest would be placed back on. Rats were monitored to ensure food and water consumption were normal without signs of distress. If signs of distress were present, usually porphyrin around the eyes or nose, the vest would be removed for approximately 24 hours to allow for recovery. Slight adjustments to this training schedule were made individually for each rat based on temperament. Vests were removed each week temporarily to check for abrasions and allow for grooming. If abrasions were found, the vest would be removed until the abrasion healed. The vests were checked once a week to ensure proper fitting and were readjusted to account for any weight gain, thereby preventing the development of abrasions. Different size vests were also used to allow for maximum comfort depending on the size of the rat.

*Protected Tether*

The connection between the percutaneous lead wires on the rat and the stimulator was achieved with a wired tether connected to a commutator (slip ring). The commutator allowed the wire tether to rotate freely without transmitting torque to the percutaneous lead wires. The tether was long enough that the rat could travel to the farthest locations in the cage but short enough that the tether never dragged across the cage. Included in this tethering rig was a strong monofilament fishing line that was attached to the commutator above the cage on one end and to hooks on the back of the vest on the other end. The monofilament line was the shortest connection between the commutator and the percutaneous leads and was designed to absorb any tension developed between the commutator and the leads thereby preventing the leads from being pulled. To protect the tether so that the rat did not chew through it, the wires and monofilament were conveyed through an 8-inch long, thin-walled, lightweight aluminum tube at the bottom of the tether. Zip ties were used to prevent the aluminum tube from sliding up the tether and exposing the wires. The tethers for control rats did not contain wires but consisted of the monofilament line protected by the aluminum tube.
